# Influence of extreme heat waves as an aggravating factor in the cause of death from cardiovascular diseases in Southeast Brazil

**DOI:** 10.1590/0102-311XEN088725

**Published:** 2026-02-06

**Authors:** Ronaldo André Castelo dos Santos de Almeida, Jéssica da Silva Santos, Letícia de Sousa Amorim, Marcelo Abrahão Strauch, Anderson Luiz Bezerra da Silveira, Emerson Lopes Olivares

**Affiliations:** 1 Universidade Federal Rural do Rio de Janeiro, Seropédica, Brasil.

**Keywords:** Climate Change, Cause of Death, Cardiovascular Diseases, Mudança Climática, Causas de Morte, Doenças Cardiovasculares, Cambio Climático, Causas de Muerte, Enfermedades Cardiovasculares

## Abstract

Extreme heat waves (HW) have intensified with climate change and represent a growing threat to cardiovascular health. Brazil, particularly the Southeast region, concentrates densely populated metropolitan areas and is highly vulnerable to the health impacts of rising temperatures. This study aimed to estimate excess mortality from cardiovascular causes associated with HW events in Southeast Brazil between 2014 and 2023. We conducted a time-series analysis using aggregated mortality data from the Brazilian Informatics Health Department and meteorological data from Brazilian National Institute of Meteorology. HW intensity was classified via the Excess Heat Factor (EHF), and excess mortality was estimated using observed-to-expected ratios. Correlation analyses between temperature and mortality from hypertension and ischemic heart disease were performed. Eleven extreme HW were identified during the study period. Mortality from cardiovascular causes coincided with HW episodes, particularly among older adults and in the largest metropolitan areas. Results indicated excess deaths during specific events, with an unusual increase observed in the winter of 2022. The correlations between mean temperature and monthly mortality were weak or negative, reinforcing the need for robust indices such as the EHF to capture health impacts of extreme heat. HW events in Southeast Brazil were associated with higher cardiovascular mortality. The findings highlight extreme heat as a relevant public health risk and reinforce the need for early warning systems, targeted mitigation strategies, and policies for urban and occupational adaptation. These results demonstrate that HW significantly aggravates cardiovascular mortality in Brazil’s most populous region.

## Abstract (pt)

As ondas de calor extremo se intensificaram com as mudanças climáticas e representam uma ameaça crescente à saúde cardiovascular. O Brasil, particularmente a Região Sudeste do país, concentra áreas metropolitanas densamente povoadas e é altamente vulnerável aos impactos do aumento das temperaturas na saúde. Este estudo teve como objetivo estimar o excesso de mortalidade por causas cardiovasculares associadas às ondas de calor extremo no Sudeste do Brasil entre 2014 e 2023. Realizou-se uma análise de séries temporais utilizando dados agregados de mortalidade do Departamento de Informação e Informática do SUS e dados meteorológicos do Instituto Nacional de Metereologia. Classificou-se a intensidade da onda de calor por meio do Fator de Calor Excesso e estimou-se o excesso de mortalidade usando razões observadas/esperadas. Realizaram-se análises de correlação entre a temperatura e a mortalidade por hipertensão e doença isquêmica do coração. Onze ondas de calor extremo foram identificadas durante o período do estudo. A mortalidade por causas cardiovasculares coincidiu com as ondas de calor, particularmente entre os idosos e nas maiores regiões metropolitanas. Os resultados indicaram excesso de mortes durante eventos específicos, com um aumento incomum observado no inverno de 2022. As correlações entre a temperatura média e a mortalidade mensal foram fracas ou negativas, reforçando a necessidade de índices robustos, como o Fator de Calor Excessivo, para capturar os impactos dos extremos de calor na saúde. Eventos de ondas de calor no Sudeste do Brasil foram associados a maior mortalidade cardiovascular. Os resultados destacam o calor extremo como um risco relevante para a saúde pública e reforçam a necessidade de sistemas de alerta precoce, estratégias de mitigação direcionadas e políticas de adaptação urbana e ocupacional. Esses resultados demonstram que as ondas de calor extremo agravam significativamente a mortalidade cardiovascular na região mais populosa do Brasil.

## Abstract (es)

Las olas de calor extremo se han intensificado con el cambio climático y representan una amenaza creciente para la salud cardiovascular. Brasil, en particular la Región Sudeste, concentra áreas metropolitanas densamente pobladas y es muy vulnerable a los efectos del aumento de las temperaturas sobre la salud. El objetivo de este estudio fue estimar el exceso de mortalidad por causas cardiovasculares asociadas a episodios de calor extremo en el sudeste de Brasil entre 2014 y 2023. Realizamos un análisis de series temporales utilizando datos agregados de mortalidad de Departamento de Informática de SUS y datos meteorológicos del Instituto Nacional de Metereología. La intensidad de las olas de calor se clasificó mediante el Factor de Exceso de Calor, y la mortalidad excesiva se estimó utilizando ratios observados-esperados. Se realizaron análisis de correlación entre la temperatura y la mortalidad por hipertensión y cardiopatía isquémica. Se identificaron once olas de calor extremas durante el periodo de estudio. La mortalidad por causas cardiovasculares coincidió con los episodios de olas de calor, especialmente entre la población de edad avanzada y en las áreas metropolitanas más grandes. Los resultados indicaron un exceso de muertes durante eventos específicos, con un aumento inusual observado en el invierno de 2022. Las correlaciones entre la temperatura media y la mortalidad mensual fueron débiles o negativas, lo que refuerza la necesidad de índices sólidos, como el Factor de Exceso de Calor, para captar los efectos de las olas de calor extremas en la salud. Las olas de calor en el Sudeste de Brasil se asociaron con una mayor mortalidad cardiovascular. Los resultados destacan el calor extremo como un riesgo relevante para la salud pública y refuerzan la necesidad de sistemas tempranos de alerta, estrategias específicas de mitigación y políticas de adaptación urbana y laboral. Estos resultados demuestran que el calor extremo agrava significativamente la mortalidad cardiovascular en la región más poblada de Brasil.

## Introduction

Global mortality has increased in line with the increase in global temperature ^1^, and part of this is due to cardiovascular events caused by high temperatures ^2^. In different regions of the world, whether hot or milder, many climate events characterize the increase in global temperature to levels never recorded before ^3^. Hot regions such as South America ^4^, Africa ^5^, and Australia ^6^ have been severely impacted by rising temperatures over the years. Moreover, countries with mild climates have suffered heat waves (HW) with temperatures above 40ºC ^7^. Effects on the environment have directly affected people’s way of life concerning behavior ^8^, fluid consumption ^9^, tolerance to heat ^10^, biological functioning, and the body’s ability to endure this type of stress ^11^. In response to this phenomenon, the scientific community has been seeking answers to improve the population’s quality of life. Numerous studies on thermoregulation ^12^ ^,^ ^13^ ^,^ ^14^ and thermoregulation during physical exercise ^15^ ^,^ ^16^ ^,^ ^17^ ^,^ ^18^ have clarified some questions regarding the organic capacity to adapt to extreme climatic environments, including under intense heat. Cardiovascular diseases and mortality from cardiovascular diseases are greatly relevant, as they are the leading cause of death in the world ^19^ and in Brazil ^1^. Among the deaths from cardiovascular causes documented in Brazil, the occurrences in the Southeast Region contributed greatly to the high numbers in the country.

HW have been documented worldwide ^3^ ^,^ ^4^ ^,^ ^5^ ^,^ ^6^ ^,^ ^7^ ^,^ ^11^ ^,^ ^20^ ^,^ ^21^ ^,^ ^22^ ^,^ ^23^ ^,^ ^24^ and are not exclusively related to an increase in cardiovascular events but also to diabetes ^1^ and respiratory diseases ^22^. HW have impacted mortality in countries such as the United States ^21^, as well as regions such as Europe ^25^, Asia ^26^, and Oceania ^27^. Brazil houses the hottest areas in South America ^4^, as it represents 47.3% of the total South American territory ^28^ ^,^ ^29^. Cardiovascular and respiratory events are responsible for increasing mortality among older adults by 5.7% ^30^. In Brazil, around 1,800 excess deaths were related to four HW events in 2010, and 2012 in Rio de Janeiro, most linked to circulatory illnesses ^1^. Notably, projections indicate an even greater increase in the coming years ^4^.

Given this scenario, it is necessary to consider the most affected groups by HW, which are older adults ^31^ and children in early childhood ^20^, as well as the most vulnerable regions, not only due to climate change, but also due to the state of adaptation of their population to this phenomenon. Organic adaptation to climate events is when the individual faces the stress caused by HW with the least possible impact on their health. Adaptation and acclimation strategies are widely used in the sports environment in search of performance ^15^ ^,^ ^18^ ^,^ ^32^ ^,^ ^33^ ^,^ ^34^ ^,^ ^35^, but they have also been widely debated as a strategy to promote quality of life ^36^ ^,^ ^37^ ^,^ ^38^ ^,^ ^39^ ^,^ ^40^ ^,^ ^41^.

The originality of this study lies in its combination of a metric still rarely used in Brazilian research, focusing on densely populated tropical urban areas, and directing the analysis to specific causes of cardiovascular mortality in a recent and comprehensive historical series (2014-2023). We seek to contribute to a broader understanding of the impacts of heat waves in the Brazilian context, as well as with comparable evidence from the international scenario.

Based on the above considerations, this study shows an analysis of the impact of HW on mortality from cardiovascular complications in the Southeastern region of Brazil from 2014 to 2023, based on data from the four most populous metropolitan areas. This study aimed to estimate excess mortality from cardiovascular causes during heat waves in the Southeast Region of Brazil. By characterizing these events and their health effects, the results may contribute to guiding public health strategies and policies for climate adaptation.

## Methods

This study’s design does not enable causal inference. The analyses are based on aggregate mortality and meteorological data, combined with descriptive methods, which are appropriate for identifying temporal patterns and associations, but not for establishing direct cause-and-effect relationships. The results should be interpreted as indicative of correlations between extreme heat events and cardiovascular mortality, rather than as evidence of causal mechanisms at the individual level.

### Target population

Brazil has a population of 203,080,756 inhabitants ^28^. The Southeast Region corresponds to 42% of the Brazilian population and has an area of 924,558,381km^2^, with three of the most populous states in the country, São Paulo, Minas Gerais, and Rio de Janeiro. The Southeast Region has 84,840,113 inhabitants, and is in an area with a predominantly tropical climate. This region also faces, year after year, the climate changes to which the world is currently subjected. Life expectancy, which in 2022 was 75.5 years ^42^, may suffer a reduction after so many extraordinary and extreme climate events. In this study, we considered the four metropolises of the Southeast Region, namely: Rio de Janeiro, São Paulo, Belo Horizonte, and Vitória.

The population of each state is 44,112,238 (São Paulo), 20,539,989 (Minas Gerais), 16,055,174 (Rio de Janeiro), and 3,833,712 (Espírito Santo).

### Data analyzed

Table 1 shows the average daily temperature data between 2014 and 2023 that were obtained from the meteorological stations made available by the Brazilian National Institute of Meteorology (INMET, acronym in Portuguese; https://portal.inmet.gov.br/). Regarding temporal scale, note that the study relies on observational data from meteorological stations, which limits the available time series. The choice of the 2014-2023 period was based on data consistency and availability across the metropolitan areas analyzed. All meteorological stations are located inside the urban core of each metropolitan area. The World Meteorological Organization (WMO) ^43^ recommends a threshold of 20% for missing data. The meteorological stations selected in the metropolitan areas were Mirante (SP-A701), Vila Militar (RJ-A621), Pampulha (BH-A521), and Vitória (ES-A621). Stations were chosen based on those metropolitan areas with complete data records in the selected period. No significant differences were observed in missingness between cold and warm months, and missing data are spread throughout the entire historical series.

**Table 1:** Heat waves occurrence between 2014 and 2023 in Southeast Brazil.

Metropolitan areas	2014	2015	2016	2017	2018	2019	2020	2021	2022	2023
Rio de Janeiro										
LIHW	7	11	5	0 *	9	7	13	11	7	6
SHW	13	5	7	2	8	7	8	9	14	5
EHW	0	0	0	0	0	0	0	2	1	0
São Paulo										
LIHW	7	7	8	12	6	6	9	10	8	1
SHW	9	9	12	6	9	8	10	11	13	0 *
EHW	0	0	1	0	0	0	0	1	0	0 *
Belo Horizonte										
LIHW	10	10	8	6	13	5	13	9	4	7
SHW	12	6	5	5	6	7	10	7	7	4
EHW	0	0	0	1	0	0	2	1	1	1
Vitória										
LIHW	7	5	7	7	9	6	0 *	1 *	4	10
SHW	7	3	7	3	8	4	0 *	0 *	7	6
EHW	0	0	0	0	0	0	0 *	0 *	0	0

EHW: extreme heat wave; LIHW: low-intensity heat wave; SHW: severe heat wave.

Note: some daily average temperatures were not available. EHW are shown in red.

* Periods in which no meteorological data was collected.

Monthly mortality data from the Brazilian Health Informatics Department (DATASUS, acronym in Portuguese) for the 2014-2023 period were provided by the Tabnet database of the DATASUS platform (https://datasus.saude.gov.br/informacoes-de-saude-tabnet/). Mortality data categorized by ICD-10 (International Classification of Disease - 10th revision) chapter included death by diseases of the circulatory system (Chapter IX). Those data were disaggregated by year/month of death, metropolitan area/RIDE, deaths by residence, and all municipalities within each metropolitan area were included, adding up to 9,357,684 deaths analyzed (2014-2023). Population data were obtained via the 2022 *Demographic Census*, released on June 28, 2023, by the Brazilian Institute of Geography and Statistics (IBGE, acronym in Portuguese) ^44^. A second analysis was performed by changing the search argument “ICD-10 Chapter” to “ICD-10 Group” hypertensive diseases and ischemic heart diseases.

### Risk analysis

A correlation analysis between the variables was conducted to verify how much the abrupt increases in temperature in each period have been influencing the population’s mortality rate and whether cardiovascular causes were impacted by HW, thus directly influencing overall mortality. The GraphPad Prism program, version 10.3.1 (https://www.graphpad.com/) was used to conduct the analysis. The test choice was based on data distribution. When normality and linearity were assumed, Pearson’s correlation coefficient was used. When data distribution was not normal, Spearman’s correlation coefficient was used.

### Characterization of HW

There is no global consensus on a definition of a HW, although it is generally considered simply a prolonged period of extreme heat, which leads research institutions (U.S. National Weather Service, U.S. National Oceanic and Atmospheric Administration, U.K. Met Office, and the WMO) to describe these events in similar but distinct ways ^45^. Generally, heat waves are a period of excessively hot weather, which may be accompanied by high humidity ^6^. Climate scientists define heat waves as prolonged episodes of abnormally high temperatures ^46^.

We classify the intensity according to the metric used previously ^47^. This methodology is based on the average temperature of three consecutive days to determine an index (Excess Heat Factor - EHF) that represents the intensity of a HW. If the daily mean temperature (DMT) averaged over the three-day period (TDP) is higher than the climatological 95th percentile for DMT (hereafter T_95_), then the TDP and each day within it are deemed to be in HW conditions. Hence, T_i_ denote the DMT calculated in this way as the average of the maximum and the minimum that occur in 24 hours. Further, T_95_ denote the 95th percentile of this DMT calculated across 2014-2023. The two variables that make up the EHF calculation are EHI_sig_ and EHI_accl_.

EHI_sig_ = (T_i_ + T_i+1_ + T_i+2_) / 3 - T_95_(1)

and:

EHI_accl_ = (T_i_ + T_i+1_ + T_i+2_) / 3 - (T_i-1_ + ... + T_i-30_) / 30 (2)

EHF is calculated as a product of these two indices:

EHF = EHI_sig_ x max (1, EHI_accl_) (3)

The duration of the HW comprises those days for which the significance index is positive, whether those days individually exceed T_95_ in their DMT. If a TDP has a positive EHF, then all the days within the TDP are HW days. When EHI_accl_ is positive, there is a deficit in acclimatization to the average temperature of the previous 30 days. An isolated hot day with DMT > T_95_ is not sufficient for a HW. The classification of HW intensity was obtained by considering the 85th percentile of all the positive EHF values (EHF_85_) as a threshold. Days with negative EHF are not considered for calculating EHF_85_. According to Nairn & Fawcett ^47^, HW can be classified as low intensity HW (LIHW: EHF < EHF_85_), severe HW (SHW: EHF > EHF_85_), and extreme HW (EHW: EHF > 3x EHF_85_).

### Excess mortality estimates

HW-related excess mortality was estimated based on a method previously used ^1^ ^,^ ^30^ to estimate the burden of heat-related mortality, the observed-to-expected deaths (O/E) ratio. This method enables one to compare directly the number of deaths observed during short periods, with the average number of deaths in similar periods, so values higher than 1 mean excess mortality, as follows:

(O/E)_ij_ = M_ij_/(M_i1_ + M_i2_ + ... + M_ij +1_ ... + M_i,k-1_ + M_i,k_/(k - 1)) (4)

Considering the ith HW that occurred in the jth year, M_ij_ is the observed number of deaths over all HW days. Expected mortality is calculated considering the average number of deaths over periods of the same duration using the same consecutive days of the year in the previous (M_i1_, M_i2_, ..., M_i,j-1_) and subsequent (M_i,j+1_, ..., M_i,k-1_, M_i,k_) years, ranging from 1 (in this case representing the year 2014) to k (2023).

### Statistics

The relationship between season and the observed number of deaths was examined using scatter plots between monthly mean temperature and monthly mortality levels. To determine a possible correlation between seasons and mortality from cardiovascular causes, we selected deaths from circulatory system problems and, to be more specific, deaths from arterial hypertension and from ischemia. Data were analyzed based on the average monthly temperature, thus seeking to verify whether there was a correlation between monthly mortality and the cause.

We performed the correlation analysis of EHF with daily deaths due to arterial hypertension (HBP) and ischemia (IQ). Considering that conditions tend to exacerbate the health conditions of vulnerable individuals suffering from circulatory illnesses, we conducted a regression analysis, also considering the mortality associated with this group of diseases.

## Results

### Relationship between mortality and season

**Figure 1:**
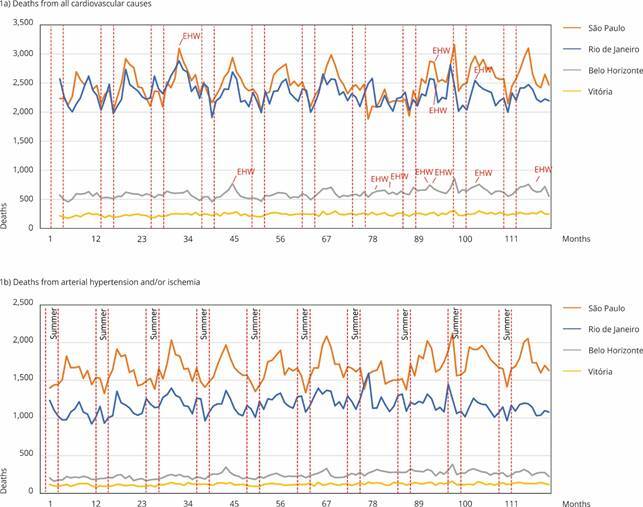
Mortality from cardiovascular causes or arterial hypertension and/or ischemia 2014-2023.

Source: prepared by the authors, based on mortality data from the Brazilian Mortality Information System (SIM).

Data on total deaths (R_TD_) and deaths by hypertension and ischemia (R_HBP and IQ_) showed low correlation between number of deaths and high temperatures in the four capitals (São Paulo: R_TD_ = -0.624, R_HBP and IQ_ = -0.687; Rio de Janeiro: R_TD_ = -0.418, R_HBP and IQ_ = -0.322; Belo Horizonte: R_TD_ = -0.418, R_HBP and IQ_ = -0.490; Vitória: R_TD_ = -0.423, R_HBP and IQ_ = -0.397). Figure 1 shows that the mortality values are lower compared to the remaining months of each year, except for the summer of 2022, in which the highest mortality rates were observed.

Except for the summer of 2022, the other nine years analyzed had lower mortality rates in absolute values. The correlation between the monthly average temperatures and total monthly mortality, mortality due to HBP or IQ was moderate and negative in São Paulo (total deaths: R = -0.6245; HBP and IQ deaths: R = -0.6862). The other metropolitan areas had a negative and very weak correlation. Therefore, different criterion is needed to assess the occurrence of HW. High temperature for a single day is not enough to determine a HW. According to what we can observe in this model, the relationship between EHI_sig_ and EHI_accl_ will determine this phenomenon.

According to the determination method of HW ^47^, we could observe the occurrence of 11 EHW in the Southeast Region between 2014 and 2023. Table 1 describes these data in detail.

From 2014-2023, considering the four metropolitan areas, 11 EHW were determined. The observed HW were in 2016 (São Paulo, June 9-13), 2017 (Belo Horizonte, June 2-7), 2020 (Belo Horizonte, May 27-29 and August 21-23), 2021 (Rio de Janeiro, August 18-20 and August 27 to September 1; São Paulo, August 27 to September 1; Belo Horizonte, August 1-4 and 28 to 30), 2022 (Rio de Janeiro, June 13-15; Belo Horizonte, May 17-20), 2023 (Belo Horizonte, July 3-6).

The results describe temporal patterns between EHW events and cardiovascular mortality in Southeast Brazil. Periods of excess heat coincided with higher mortality, particularly among older adults and in metropolitan areas with greater population density. The analysis of observed versus expected mortality (O/E) indicates an increase during specific heatwave episodes, suggesting an association rather than a direct causal effect.

## Discussion

HW have been recognized as one of the most critical health threats related to climate change, with a consistent increase in cardiovascular mortality on a global scale ^2^. Studies indicate that each 1ºC increase in temperature is associated with a ~2% increase in cardiovascular mortality ^2^. Furthermore, global projections indicate that tropical and subtropical countries, such as Brazil, are among the most vulnerable to mortality from extreme heat ^3^.

EHF has been increasingly adopted as a practical indicator to identify and classify HW for public health surveillance, since it is based on thermal deviations and population acclimatization over recent periods ^47^. The use is justified by the growing evidence linking heat exposure to increased cardiovascular morbidity and mortality ^22^ ^,^ ^48^ ^,^ ^49^, including recent findings in Rio de Janeiro and other Brazilian urban areas ^1^ ^,^ ^22^ ^,^ ^30^. However, EHF has important limitations, as it assumes homogeneous acclimatization and does not incorporate physiological or biometeorological responses that are critical for assessing vulnerability, particularly among older adults and chronically ill populations ^10^ ^,^ ^12^ ^,^ ^13^ ^,^ ^14^. Thus, although EHF is a valuable population-level tool, its application should be accompanied by cautious interpretation and complemented with alternative approaches that integrate human physiology to better guide adaptive health policies.

The increase in global temperatures resulting from climate change ^50^ constitutes a serious threat to public health. Analysis of causes of death based on data from DATASUS demonstrates underreporting of problems related to heat stress (HS), since the cause of death are defined based on events observed when medical care is provided. When there is underreporting of the primary cause, the problem is underestimated. According to previous studies, environmental conditions and high temperatures already compromise the productivity and health of thousands of workers ^51^.

The HS concept should be applied with greater precision, since it represents a well-defined physiological condition characterized by the imbalance between heat production and heat dissipation ^10^ ^,^ ^12^ ^,^ ^17^. This imbalance can lead to thermal overload and compromise thermoregulatory mechanisms, which increases health risks. Importantly, HS does not affect only occupational groups exposed to high temperatures, but also children, older adults, pregnant women, and people with preexisting comorbidities, who have limited physiological reserve to cope with thermal extremes ^2^ ^,^ ^22^ ^,^ ^52^. During HW, the intensity and duration of HS are amplified, which increases the vulnerability of these groups and underscores the importance of using the term accurately in public health and epidemiological contexts ^1^ ^,^ ^30^ ^,^ ^52^.

Many studies have described climate change around the world ^10^ ^,^ ^25^ ^,^ ^26^ ^,^ ^27^ ^,^ ^53^ ^,^ ^54^ ^,^ ^55^. In Brazil, specifically in the Southeast Region, previous studies have shown higher mortality rates during HW periods ^1^ ^,^ ^19^ ^,^ ^22^. These studies described the occurrences mainly in the first 15 years of the 2000s. We found that the increase in global temperatures also affected Brazil and brought thermal discomfort as an aggravating factor in the quality of life of the population in the Southeast. According to our analysis, 11 more significant HW occurred between 2014 and 2023. In January, February, and March 2022, we observed an exponential increase in deaths in São Paulo, Belo Horizonte, and Rio de Janeiro, but not in Vitória. This was a period immediately following the COVID-19 pandemic, and we were unable to isolate the post-pandemic effect in our analysis. The high mortality observed in winter is not related to temperatures above the normal range. These HW episodes were defined as periods of three or more consecutive days with EHF positive values. Nine EHW events were correlated with mortality in this same period. Deaths from cardiovascular causes were the leading cause of death, and within this category, HBP and IQ led the specific causes.

Analysis of population subgroups suggests a greater impact of HW on older adults and individuals with chronic conditions ^48^ ^,^ ^56^. This pattern has also been described in Asian megacities, where mortality among older adults increased significantly during HW ^31^. Furthermore, in the Brazilian context, social inequalities accentuate vulnerability to HW, with a higher risk in peripheral urban areas with less infrastructure ^30^.

The highest total mortality rates were observed in winter. Although summer have records of higher temperatures, a decisive analysis to correlate deaths and high temperatures is not possible without considering the level of acclimatization of the population and the history of temperature records before a specific period of mortality.

An initial analysis was not sufficient to determine the cause-and-effect relationship in the causes of death. Although the highest temperatures are recorded in the summer, mortality in the Southeast Region was higher in the winter. Therefore, it was not possible to analyze whether heat waves affect population mortality. The results showed that mortality incidence levels tend to increase significantly when these conditions are reached. The best fit of the model was obtained using the EHF as a predictor, considering only deaths caused by cardiovascular diseases.

The EHF is a useful tool for public health surveillance because it enables the identification of HW events based on thermal deviations and population acclimatization over recent weeks. It is useful for monitoring and alerting systems due to its ease of use and reliance on publicly accessible meteorological data. The index does not include clinical responses or direct physiological indicators, which are essential for comprehending health risks at the individual level, so it has inherent limitations. EHF may understate vulnerability in particular groups, especially older adults or people with preexisting conditions, in contrast to indices like the Heat Index, WBGT (wet-bulb globe temperature), or UTCI (universal thermal climate index), which incorporate biometeorological or physiological parameters.

Although this short series (2014-2023) is adequate to capture interannual variations and extreme events, it is insufficient to assess long-term trends associated with climate change. For this reason, the results should be interpreted within the scope of short-term variability rather than as evidence of long-term climatic shifts. A relevant aspect was the observation of high mortality rates during winter. This result highlights the complexity of the phenomenon, which may be related to the population’s thermal acclimatization and seasonal interactions with respiratory diseases ^52^ ^,^ ^57^ ^,^ ^58^. Studies highlight that acclimatization has fixed physiological limits, which restrict human adaptation to temperature extremes ^10^. Additionally, defining HW using robust metrics, such as the EHF, has been proposed to better characterize the intensity and severity of these events ^47^.

Looking at this problem in the future creates an expectation of increased exposure to HS due to climate change, which tends to increase average temperatures ^50^, especially in tropical regions such as Brazil. In addition to the increase in average temperatures, climate change generates an expectation of increased frequency and intensity of HW ^55^ and directly impacts the worsening of working conditions, increasing the time spent working in unhealthy conditions, especially in external and internal environments where there is no control over environmental conditions. In addition to constantly changing climate conditions ^55^, population growth and disorderly urbanization in large urban centers can increase the number of workers in informal and vulnerable sectors, such as construction and street trading. In these sectors, the lack of monitoring, adequate infrastructure, and public policies to mitigate the effects of heat exposure tends to aggravate the problem. Disorderly population growth creates an unhealthy environment in homes in the most vulnerable areas.

The causes of the impacts on public health are multifactorial; among the most severe are the projected increase in heat-related diseases such as heat stroke ^49^, dehydration ^59^, cardiovascular diseases ^2^ ^,^ ^49^, and kidney diseases ^59^. The public health system may be overloaded in the most affected regions, especially in HW events.

The impact on public health is due to the direct effect of the population’s exposure to heat, but also directly to economic productivity, which will also be reflected in increased spending on health systems. Previous studies indicate that extreme heat can reduce workers’ physical productivity in different work activities ^60^. The most affected areas include agriculture ^54^, mining ^61^, and construction ^53^.

The predicted greater demand for health care from the population in the face of adverse weather events also increases the demand for adaptations in labor laws to include updated heat exposure limits, mandatory breaks between work sessions, structural adaptations in the work environment, and personal protective equipment. In this sense, advances in technology enable the development of equipment that facilitates heat exchange with the environment ^62^, body temperature monitoring ^62^, and artificial microclimates ^63^. Depending on the labor need, strategies such as teleworking and process automation are alternatives that favor work in environments with lower levels of HS ^63^.

From an economic point of view, municipalities located in regions with high temperatures and less public investment tend to suffer more from the effects of climate change. Groups of workers with lower incomes and worse working conditions will be less predisposed to acclimatization and protection, exacerbating differences between social and public health groups. The political consequences of prospects resulting from environmental HS will influence the formulation of public policies.

The authorities responsible for decision-making must work to update labor legislation to address the issue of HS and create specific and appropriate standards to the reality of the population. In extreme cases, HS can be considered an occupational risk, and the possibility of adding labor rights for possible unhealthiness should be considered. Considerations such as expanding worker health surveillance programs to address HS, increasing the financial resources of the public health system for application in more vulnerable regions, and including specific procedures for treating cardiovascular events resulting from HS should be part of the repertoire of tools to combat deaths caused by this phenomenon. The possibility of creating specific indicators that point to the primary cause of a cardiovascular event can contribute to reducing vulnerability to extreme HS.

## Conclusion

Our study characterized extreme heat in the capitals of Southeastern Brazil between 2014 and 2023 and investigated its impact on cardiovascular mortality. Eleven extreme heat events were identified, with a consistent increase in mortality, including in winter months, reinforcing the complexity of the phenomenon and the influence of factors such as thermal acclimatization and seasonal conditions. The findings confirm that extreme heat constitutes a significant public health risk, with a greater impact on vulnerable groups, such as older adults and individuals with chronic diseases. Furthermore, social and urban inequalities stand out, intensifying the effects of extreme heat on the population. From a public health perspective, the results reinforce the need to implement early warning systems, mitigation measures targeting vulnerable groups, and urban and occupational adaptation policies to reduce the adverse effects of extreme heat. Understanding the possibilities for protection against HS depends on multidisciplinary efforts that involve everything from strategic political planning of public policies to infrastructure supporting the population, and the delivery of care by healthcare professionals. Considering the projections of increased frequency, duration, and intensity of HW in the coming decades, a seemingly inevitable problem, the results of this study can serve as a basis for local epidemiological surveillance strategies, prevention of avoidable deaths, and formulation of public policies that integrate health and climate change.

### Limitations

The use of the EHF as the primary metric for identifying heat waves and analyzing observed/expected excess mortality (O/E) is consistent with the study’s aim. However, EHF assumes homogeneous acclimation based on the previous 30 days, which may underestimate risks in vulnerable groups such as older adults or individuals with chronic diseases. Furthermore, while the EHF are independent of more complex meteorological variables (e.g., humidity, solar radiation), it does not directly incorporate physiological or biometeorological factors. Recent literature highlights the potential of more health-sensitive indices, such as UTCI, WBGT, or the Heat Index, and these approaches could be considered in future research, as well as inserting a description such as age and sex data to better characterize the most affected groups.

Although issues related to informal work and labor legislation are highly relevant in the context of heat exposure, they were not directly addressed by the data analyzed in this study. These aspects should therefore be interpreted as broader perspectives for future research and policy development, particularly in exploring how labor regulations and working conditions may influence population vulnerability during extreme heat events.

## Data Availability

The research data are available upon request to the corresponding author.
